# Supplementary material for: Total and endothelial cell-derived cell-free DNA in blood plasma does not change during menstruation
Source: PLoS One. 2021 Apr 26;16(4):e0250561. doi: 10.1371/journal.pone.0250561 (PMC8075187; doi:10.1371/journal.pone.0250561)
Supplement: S1 Table — (DOCX) [file pone.0250561.s001.docx]

**S1 Table. Demographic of 40 healthy female volunteers.**

| **Pair** | **Age** | **BMI^a^** | **Current smoking (Y/N)** | **Interval time between NM and M phases blood collection (days)** | **Day(s) since beginning of menstruation** | **Oral contraceptive use (Y/N)** |
| --- | --- | --- | --- | --- | --- | --- |
| 1 | 27 | 21.5 | N | 15 (1 cycle) | 1 | N |
| 2 | 26 | 25.7 | N | 7 (1 cycle) | 1 | Y |
| 3 | 21 | 19.8 | N | 25 (1 cycle) | 1 | N |
| 4 | 22 | 20.7 | N | 25 (1 cycle) | 1 | N |
| 5 | 26 | 18.7 | N | 52 (2 cycles) | 2 | N |
| 6 | 21 | 19.9 | N | 15 (1 cycle) | 2 | Y |
| 7 | 22 | 25.3 | N | 41 (2 cycles) | 1 | N |
| 8 | 30 | 20.7 | N | 58 (2 cycles) | 1 | Y |
| 9 | 23 | 18.6 | N | 8 (1 cycle) | 2 | Y |
| 10 | 21 | 25.7 | N | 12 (1 cycle) | 1 | N |
| 11 | 32 | 23.9 | N | 8 (1 cycle) | 1 | N |
| 12 | 36 | 24.3 | N | 17 (1 cycle) | 1 | N |
| 13 | 21 | 20.8 | N | 78 (2 cycles) | 1 | Y |
| 14 | 36 | 16.6 | N | 14 (1 cycle) | 1 | N |
| 15 | 26 | 19.7 | N | 17 (1 cycle) | 1 | N |
| 16 | 36 | 25.6 | N | 32 (2 cycles) | 2 | N |
| 17 | 33 | 20.2 | N | 14 (1 cycle) | 2 | N |
| 18 | 23 | 24.3 | N | 40 (2 cycles) | 2 | N |
| 19 | 34 | 23.1 | Y | 20 (1 cycle) | 1 | N |
| 20 | 47 | 23.3 | N | 37 (1 cycle) | 2 | N |
| 21 | 29 | 20.7 | N | 16 (1 cycle) | 1 | N |
| 22 | 22 | 21.7 | N | 13 (1 cycle) | 1 | N |
| 23 | 21 | 17.9 | N | 47 (1 cycle – irregular cycle) | 1 | N |
| 24 | 38 | 23.0 | N | 15 (1 cycle) | 1 | N |
| 25 | 25 | 22.0 | N | 14 (1 cycle) | 1 | N |
| 26 | 30 | 22.9 | N | 14 (1 cycle) | 1 | N |
| 27 | 31 | NA^b^ | N | 42 (1 cycle - contraception) | 2 | Y |
| 28 | 33 | 22.8 | N | 14 (1 cycle) | 1 | N |
| 29 | 24 | 23.6 | N | 14 (1 cycle) | 1 | Y |
| 30 | 40 | 21.7 | N | 10 (1 cycle) | 2 | N |
| 31 | 26 | 22.9 | N | 20 (1 cycle) | 1 | Y |
| 32 | 39 | 21.5 | N | 11 (1 cycle) | 1 | N |
| 33 | 49 | 23.2 | N | 17 (1 cycle) | 1 | N |
| 34 | 28 | 23.2 | N | 15 (1 cycle) | 2 | Y |
| 35 | 34 | 20.6 | N | 69 (3 cycles) | 2 | Y |
| 36 | 43 | 20.4 | N | 16 (1 cycle) | 2 | N |
| 37 | 27 | 19.6 | Y | 48 (2 cycles) | 1 | N |
| 38 | 42 | 24.6 | N | 15 (1 cycle) | 1 | N |
| 39 | 34 | 22.6 | N | 18 (1 cycle) | 2 | N |
| 40 | 31 | 27.6 | N | 15 (1 cycle) | 2 | Y |

^a^BMI <18.5: underweight; 18.5 to <25: normal; 25 to <30: overweight as defined by Centers for Disease Control and Prevention.

^b^NA: Not available
