# Supplementary figures and images for: Total and endothelial cell-derived cell-free DNA in blood plasma does not change during menstruation
Source: PLoS One. 2021 Apr 26;16(4):e0250561. doi: 10.1371/journal.pone.0250561 (PMC8075187; doi:10.1371/journal.pone.0250561)

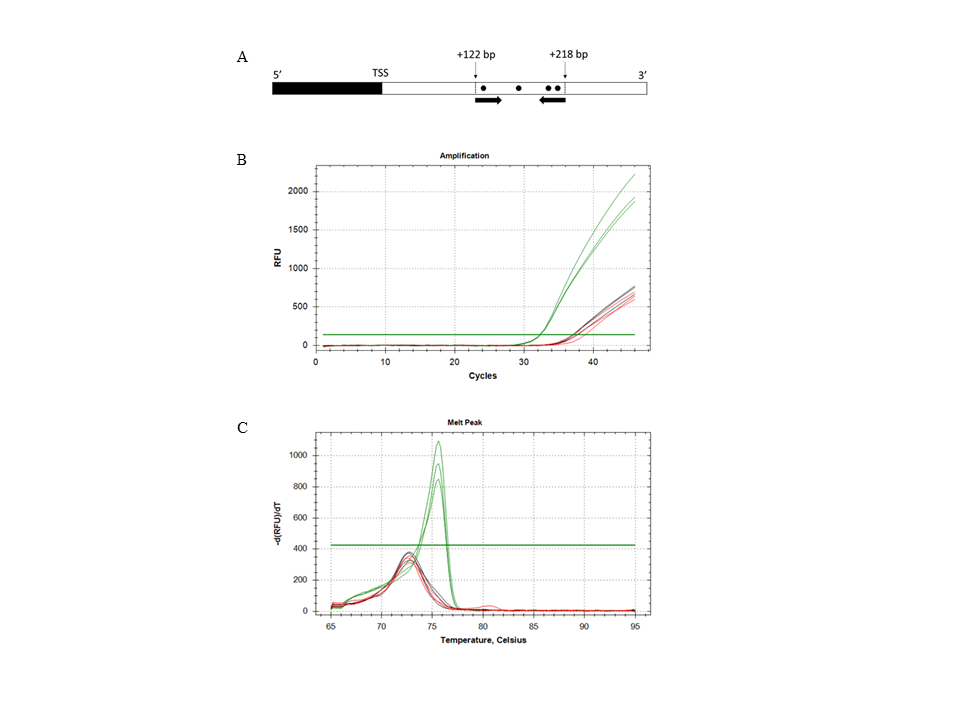

Supplement: S1 Fig — (A) A schematic of primer location in the CDH5 region spanning from +122 bp to +218 bp. CpG mismatches are shown as •. The arrows indicate forward and reverse primers. qPCR amplification (B) and melt curve (C) plot of CDH5 primer selectivity and specificity in 1 ng of in-vitro unmethylated (green) and methylated DNA (red) set with NTC (black) as control. (TIF) [file pone.0250561.s002.tif]

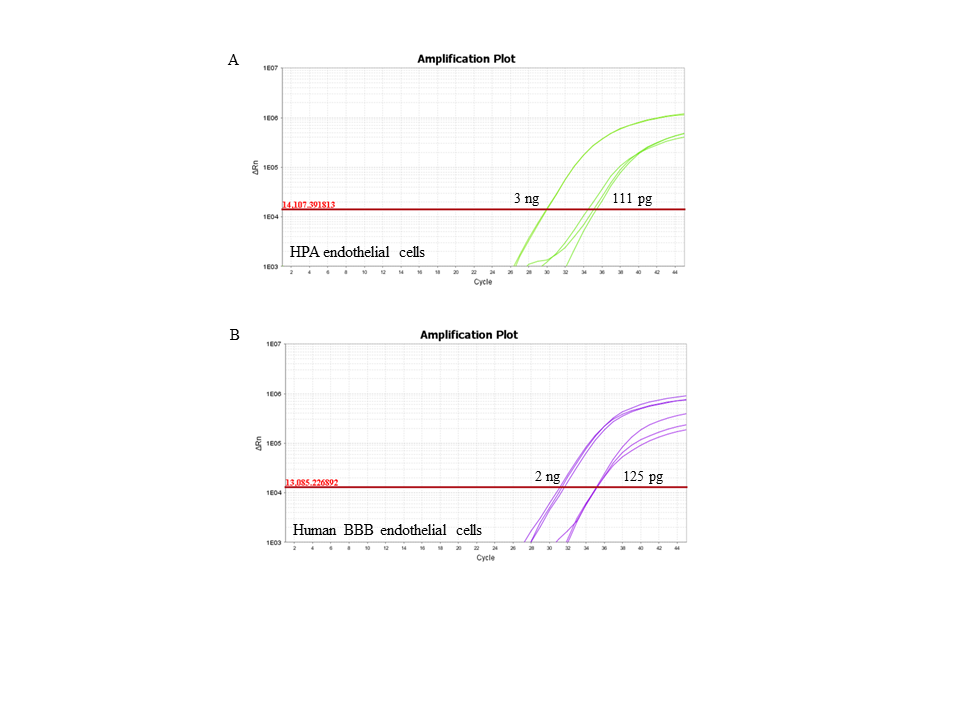

Supplement: S2 Fig — qPCR amplification plot of CDH5 in (A) human aortic endothelial cells (3 ng and 111 pg) and (B) blood brain barrier endothelial cells (2 ng and 125 pg) as primer validation. (TIF) [file pone.0250561.s003.tif]
